# Surgical treatment of postoperative intractable bile leakage after liver tumor surgery in children

**DOI:** 10.3389/fped.2023.1110042

**Published:** 2023-05-15

**Authors:** Jianyu Han, Hong Qin, Wei Yang, Haiyan Cheng, Xiaofeng Chang, Zhiyun Zhu, Jun Feng, Shen Yang, Yajun Chen, Huanmin Wang

**Affiliations:** ^1^ Department of Surgical Oncology, Beijing Children’s Hospital, Capital Medical University, National Center for Children’s Health, Beijing, China; ^2^ Department of General Surgery, Beijing Children’s Hospital, Capital Medical University, National Center for Children’s Health, Beijing, China

**Keywords:** bile leakage, surgery, children, liver tumor, postoperative, bilio-cholecyst anastomosis

## Abstract

**Aim:**

To summarize systematically our six-year experience in the surgical treatment of postoperative bile leakage after liver tumor surgery in children, and explore its reoperation approach and treatment effect.

**Methods:**

The clinical data of 6 patients with postoperative bile leakage cured by surgery from January 2016 to January 2022 were reviewed retrospectively.

**Results:**

Among the six pediatric patients with postoperative bile leakage cured by surgery, four were male (67%) and two were female (33%). All patients underwent complex segmentectomy. The median time to bile leakage was 14 days (range, 10 to 32), and the daily drainage volume was stable from 170 ml to 530 ml per day. After conservative treatment failed, four patients received biliary-enteric anastomosis (patients 1, 3, 4, and 6), and two patients received bilio-cholecyst anastomosis (patients 2 and 5). All six patients were successfully treated with reoperation, and five patients were alive and without recurrence, while one patient was lost to follow-up due to abandoned treatment.

**Conclusion:**

Our study suggests that surgery is a reliable and effective treatment for postoperative intractable bile leakage in children undergoing complex segmentectomy. Bilioenteric anastomosis is the most common technique for bile leakage, and bilio-cholecyst anastomosis is a feasible and effective surgical approach. These findings have important implications for the management of postoperative complications in pediatric patients undergoing complex segmentectomy.

## Introduction

Bile leakage is one of the most common complications after liver tumor surgery (1). Despite a significant decrease in the overall surgical complication rate in hepatic tumor resections, the rate of bile leakage has not changed, with an incidence of 5%–12.5% (2) in children. Bile leakage can lead to postoperative morbidities, such as abdominal abscesses, longer hospital stays, delayed removal of abdominal drains, and higher postoperative mortality due to liver failure. However, the experience of reoperation surgical treatment for children with intractable bile leakage is limited. Therefore, it is imperative to explore surgical treatment approaches and increase surgical experience for children with refractory bile leakage after liver tumor surgery. To address these issues, we retrospectively analyzed patients who received reoperation for intractable bile leakage after liver tumor surgery at our center.

The study aimed to evaluate the amount of bile leakage and summarize the treatment outcomes of reoperation, proposing a surgical approach for postoperative bile leakage in children.

## Patients and methods

### Patients

We conducted a retrospective review of medical records from Beijing Children's Hospital, analyzing 20 cases of postoperative bile leakage following hepatic tumor resections between January 2016 and January 2022. Of the 155 liver tumor operations performed during this period, 117 involved hepatoblastoma while the remainder included liver metastases, focal nodular hyperplasia, hamartoma, hepatocellular carcinoma, and vascular malformation. (Table 1) Patients who underwent hepatic resections with extrahepatic biliary resection and reconstruction during the first operation were excluded from the study.

**Table 1:** Patients, surgical procedures and indications for surgery.

	Without bile leakage	Conservative treatment for bile leakage	Surgical treatment for bile leakage
**Patients**			
Total number of patients	135	14	6
Male	70	6	4
Female	65	8	2
Median age during surgery (months)	31 (range: 3–199)	29 (range: 9–138)	28.5 (range: 5–137)
**Indications for surgery**			
Hepatoblastoma	101	12	4
Liver metastases	19	0	0
Focal nodular hyperplasia	8	2	1
Hamartoma	5	0	0
Hepatocellular carcinoma	1	0	1
Vascular malformation	1	0	0
**Surgical procedures**			
Resection of 1–2 segment(s)	97	5	1
Resection of 3–4 segments	35	8	3
Resection of ≥5 segments	3	1	2

To identify risk factors for postoperative bile leakage, we analyzed three types of variables: Patient variables (sex, age, and serum level of alpha-fetoprotein), Surgical variables (surgical procedures, indications for liver resection, and bile leakage drainage), and Tumor variables (tumor diameter, diagnosis of liver tumor, and reoperation time). We analyzed the characteristics of patients who required reoperation and summarized the indications for reoperation.

## Methods

### Definition of bile leakage

Many different definitions of bile leakage have been proposed before, with the final uniform definition and grading of bile leakage established by the International Study Group of Liver Surgery (ISGLS) in 2011 (3). Bile leakage was defined as a bilirubin concentration in the drain fluid at least 3 times the serum bilirubin concentration on or after postoperative day 3 or as the need for radiologic or operative intervention resulting from biliary collections or bile peritonitis. Intractable bile leakage was defined as bile leakage that did not improve after a drainage procedure.

### Surgical procedures

During surgery, we used microwave coagulation and bipolar to separate abdominal adhesions, and carefully identified liver anatomy and bile duct pathways. An intraoperative bile leakage test was routinely performed using diluted indocyanine green, which allowed for identification of the bile leakage site. If necessary, a stent tube was placed under intraoperative ultrasound guidance from the suspected bile leak location to confirm the site of the leakage. The approach of anastomosis was determined based on the intraoperative leakage site, and the surgical area was continuously observed, with the white gauze test (4) used to ensure no bile leak remained.

Intra-abdominal drainage catheters were routinely placed in all patients, with the drainage catheter removed when the drainage fluid was minimal and not bile-stained. Systemic antibiotics (usually second-generation cephalosporin) were routinely given during treatment (5).

## Results

We counted the number of patients with intractable bile leaks after liver tumor resection in our institution and outside hospitals. Of the six patients identified, four were male and two were female, with a median age of 28.5 months (range 5 months–137 months). Four patients were diagnosed with hepatoblastoma (HB) and received surgery after standard chemotherapy, which resulted in significant shrinkage of tumor size. One patient was diagnosed with focal nodular hyperplasia (FNH) through needle biopsy and received surgery after hepatic artery embolization. One patient had a controversial pathological diagnosis based on needle biopsy, and received primary resection. Serum alpha-fetoprotein (AFP) was significantly elevated in HB patients and decreased rapidly after chemotherapy. The largest tumor diameter ranged from 4.7 cm to 15 cm. The patients’ information is shown in Table 2.

**Table 2:** Patient information.

Patient No.	Age (months)	Gender	Diagnosis	Tumor size at diagnosis (cm)	Segment involvement in tumor resection	Surgical approach	Intraoperative bile leakage test	Intraoperative management
1	5	Female	HB	11	III,IV,V,VI,VII,VIII	Nonanatomical resection*	Negative	–
2	24	Male	HB	9.0	VI,VII	Anatomical resection	Failed	
3	17	Male	HB	4.7	II,III,IV	Anatomical resection	Negative	–
4	33	Male	HB	8.6	II,III,IV	Nonanatomical resection	Negative	–
5	137	Male	HCC	15	V,VI,VII,VIII	Anatomical resection	Positive	Intraoperative repair
6	42	Female	FNH	10	II,III,V,VI,VII,VIII	Nonanatomical resection	Negative	–

HB, hepatoblastoma; HCC, hepatocellular carcinoma; FNH, focal nodular hyperplasia; POD, postoperative day.

* Right trisectionectomy + S3 subsegmentectomy.

Five patients underwent major hepatectomy, which is defined as the resection of three or more Couinaud segments. Open surgery was performed in all cases to remove the liver tumors, and intraoperative cholangiography was employed to identify any bile leakage. In one case (patient 5), intraoperative angiography revealed methylene blue leakage in the common hepatic duct, which was subsequently repaired during the procedure. No bile leakage was detected on repeat cholangiography following suturing.

**FIGURES 1–6:**
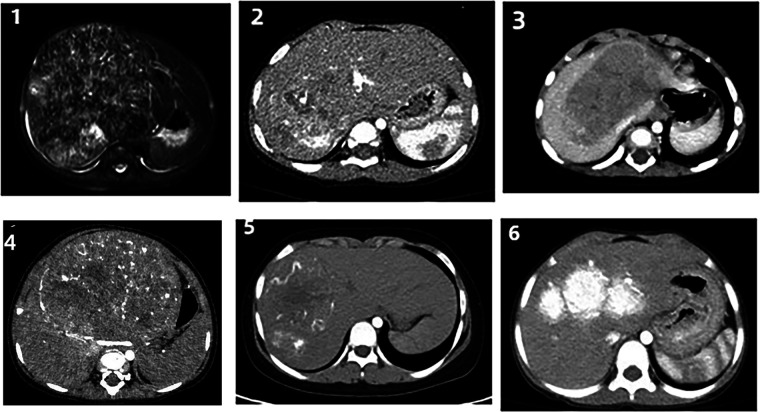
Figures 1–5 Contrast-enhanced CT images obtained at the time of diagnosis for patients 1–5, showing the presence and location of tumors in the liver. Figure 6 shows a post-interventional embolization image of patient 6, revealing successful arterial occlusion following selective arterial embolization before sugey.

Figures 1-5 Contrast-enhanced CT images obtained at the time of diagnosis for patients 1-5, showing the presence and location of tumors in the liver. Figure 6 shows a post-interventional embolization image of patient 6, revealing successful arterial occlusion following selective arterial embolization before sugey.

### Bile leakage

We identified six patients with intractable bile leakage, with a median time of diagnosis at postoperative day 14 (range: 10-32 days). Adequate drainage was initiated promptly upon confirmation of bile leak and the daily volume of bile leakage remained stable, ranging from 170 ml to 530 ml per day, exceeding 10 ml per kg of body weight per day. All patients underwent reoperation after conservative treatment had failed, with a duration ranging from 39 to 182 days after the diagnosis of bile leakage.

In our cohort of patients with refractory bile leaks, we observed that the leakage was localized to the region of the bile duct or its secondary branches, as confirmed during the reoperation.

### Postoperative course

Following the analysis of blood routine results and drainage bacterial culture, effective antibiotics were administered. For patients diagnosed with bile leakage, bile bacterial culture was routinely performed to select sensitive antibiotics based on the culture results.

Moreover, one patient (Patient 3) developed intestinal obstruction 40 days after the operation and underwent intestinal adhesion release and bile drainage.

### Management and outcomes

Initially, all six patients were managed non-surgically with measures such as ensuring adequate drainage, percutaneous abdominal paracentesis or drainage, and monitoring of liver function and ascites bilirubin levels. Four of the patients had biliary obstructive jaundice at the time of biliary leakage, as demonstrated by laboratory tests. In two of these cases, the obstruction was relieved and liver function improved with duct drainage, allowing for adequate preoperative preparation. The other two cases with persistent jaundice were promptly treated surgically.

After conservative treatment failed, all six patients underwent surgery. Four patients (patient 1, 3, 4, and 6) received biliary-enteric anastomosis, while two patients (patient 2 and 5) underwent bilio-cholecyst anastomosis. Following reoperation, all patients recovered well, with no further bile leakage. They were discharged after successful removal of the drainage tube, with the length of stay ranging from 20 to 41 days.

Despite the complexity of the surgery, the location of the bile leak was identified and repaired successfully. Five patients are currently alive, while one patient was lost to follow-up due to abandoned treatment.

## Discussion

Bile leakage is a commom complication after liver surgery, but the treatment is difficult and has a great impact on postoperative recovery and subsequent treatment (6). While children's experiences with bile leakage are mostly derived from adults, the types of diseases and treatment methods in children are unique. Despite this, few reports have been published on the surgical treatment of bile leakage in children. In our study, we observed 135 patients (87%) without bile leakage, 14 patients (9%) with conservative treatment, and 6 patients (3.8%) who required reoperation. The incidence of postoperative bile leakage after liver surgery in our center is approximately 13.3% (7), which is consistent with international reports (2).

### Risk factors and perioperative events

Previous research has shown that high-risk surgical procedures, such as those involving the major Glisson's sheath and hepatic hilum (e.g., anterior segmentectomy, central bisegmentectomy, or total caudate lobectomy), are independent predictors of postoperative bile leakage (8). In this study, the resected liver segments of the patients also included these parts. Thus, the possibility of biliary leakage is further increased in children with large liver tumors and those located in proximity to the hepatic hilum. Furthermore, the presence of bile, blood and devitalized tissues in the surgery area provides an ideal environment for bacterial growth which can lead to the development of intraperitoneal septic complications after hepatectomy (IPSCH) (9). In turn, the infection may further precipitate biliary leakage by inducing tissue necrosis (10).

It is undisputed that the drainage of intraperitoneal bile is crucial in the management of bile leakage following liver surgery. During the postoperative course, regular assessment of the presence of intraperitoneal bile is imperative, and in cases where insufficient bile drainage is noted, additional subcutaneous drains must be inserted.

The surveillance of bacteria in drained bile should be conducted frequently to prevent intraperitoneal septic complications after hepatectomy (IPSCH), and the administration of systemic antibiotics should be guided by the results of the culture of the drained bile (11). However, long-term tube drainage is susceptible to infection-related complications. Among the six patients in our study, three were found to have positive drainage cultures for bacteria (Table 3). In patient 1, the combination of extreme liver resection and IPSCH led to hemophagocytic syndrome, which ultimately responded to high-dose anti-inflammatory antibiotics and supportive therapy (12).

**Table 3:** Postoperative conditions, management, and prognosis of the patients.

Patient No.	Bile leakage day(POD)	Drainage (ml/day)	Drainage per body weight (ml/kg/day)	Drainage culture	Time from bile leak to reoperation (day)	Reoperation approach	Time from reoperation to discharge (day)
1	10	170	24.3	Pseudomonas aeruginosa	182	Biliary-enteric anastomosis	41
2	13	250–360	20.8–30	–	39	Bilio-cholecyst anastomosis	38
3	32	290	29	–	92	Biliary-enteric anastomosis	27
4	15	200	10	Staphylococcus epidermidis, Methicillin-resistant Staphylococcus	150	Biliary-enteric anastomosis	30
5	10	320–530	6.4–10.6	–	42	Bilio-cholecyst anastomosis	23
6	15	200	13.3	Stenotrophomonas baumannii, Stenotrophomonas maltophilia	150	Biliary-enteric anastomosis	20

### Features of intractable bile leakage

In this study, the occurrence of bile leakage proved intractable, manifesting 10 to 32 days after the surgical procedure. This delay contrasts with the presentation in patients who responded to conservative treatment, wherein bile leaks appeared earlier (7). Our hypothesis is that this phenomenon may be linked to delayed necrosis stemming from extensive bile duct damage, or to obstructive jaundice characterized by high pressure that eventually causes rupture of the site of leakage.

In an effort to identify the site of leakage prior to reoperation, we utilized magnetic resonance cholangiopancreatography (MRCP) in the children. However, given the inflammatory reaction and the presence of a bile leak cyst, it was difficult to obtain a definitive location. Therefore, we conducted a thorough exploratory dissection during reoperation and employed intraoperative ultrasound to precisely locate the hepatic duct as the source of the leakage.

### Surgical methods

Complicated liver surgery in children inevitably involves exposuring of major Glisson's sheath, which has been reported as an independent risk factors for bile leakage (13). During the operative procedures, devices that generate heat should be used carefully in regions near the Glisson's sheath to avoid damage to this critical anatomical structure. To preserve as much residual liver tissue as possible, three patients in this study underwent non-anatomical resections according to the Tokyo 2020 terminology of liver anatomy and resections (14) Although this may increase the possibility of small bile leakage (1), we found that in patients with intractable bile leakage requiring reoperation, the leakage site is usually close to the main biliary tract, which was not interfered with in this study.

During the reoperation, more careful separation is necessary, such as ligation of the small bile ducts around the hepatic hilum, avoiding damage to the anatomically variant bile ducts, and using intraoperative bile leakage test. The anatomical structures of the hilar region are poorly separated due to local inflammatory adhesions, which can easily damage the hepatic artery and portal vessels and lead to additional bleeding. Before reoperation, MRCP, which provides anatomic visualization of the operated biliary tract (15), is recommended to assess the extra-biliary bile duct path and the dilatation of intrahepatic bile ducts.

Given the serious consequences of bile leakage, patients requiring reoperation should be operated on carefully, and strict monitoring after surgery is necessary. New methods, such as the surgical administration of indocyanine green to improve bile leakage detection, can be adopted in the future (16).

### Indications for reoperation

In patients with intractable bile leakage, daily bile leakage can reach significant volumes. In our study, the minimum daily bile drainage volume was 170 ml. According to Vigano, interventional treatments should be considered in patients with drainage output greater than 100 ml after 10 days of bile leakage diagnosis (17). While there is no consensus regarding the optimal approach in children, conservative observation is often chosen for a month. However, surgical treatment should be considered if there is no improvement. Based on our data and experience, we recommend surgical therapy in the following cases: daily postoperative bile leakage exceeding 150 ml (or over 10 ml/kg/day) and persisting for over a month. If uncontrolled obstructive jaundice is present, more aggressive surgical measures may be necessary. Additionally, children with biliary leakage and concurrent infection are at greater risk for disease progression and may require more aggressive surgical intervention following control of the infection (18).

In addition to surgical treatment, ERCP has been shown to be an effective and minimally-invasive treatment option for bile duct injury in children (2, 19). However, it should be noted that ERCP may have limited impact on the distal common bile duct, especially when patients have developed an ischemic or iatrogenic stricture during the chronic course of the injury. While new treatment modalities may be explored in the future, it is important to carefully consider the appropriate treatment approach for each individual case.

### Discussion of reoperation approach

For patients who have failed conservative treatment, a thorough evaluation of the patient is necessary before surgery, and a surgical plan should be formulated accordingly. Bilioenteric anastomosis, a traditional and common surgical method, is a viable option for patients with explicit bile leakage site. During the operation, it is essential to carefully dissect the structure of the proximal and distal bile duct around the leakage area, and separate as much bile duct length as possible for anastomosis. Full anastomosis of the bile duct to the jejunal mucosa is necessary (20), and the suture between the bile duct and the jejunal intestine should be firm and reliable to prevent recurrent bile leakage. In our study, we conducted this surgical procedure on four patients who experienced a prolonged duration between the onset of bile leakage and reoperation, resulting in gallbladder atrophy and non-functionality that precluded bilioenteric anastomosis. The outcomes were favorable, with complete healing of the bile leakage observed in all cases.

**Figure 7:**
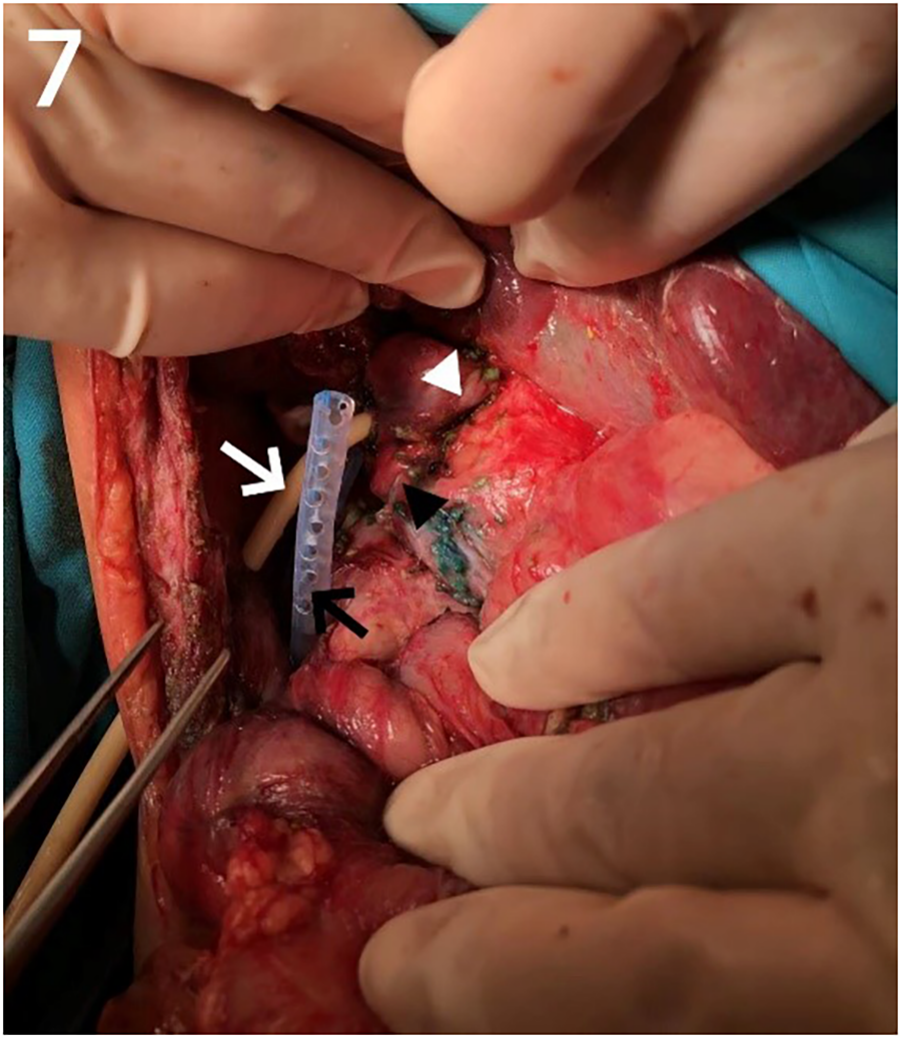
Bilio-cholecyst anastomosis images. White triangle: anastomosis; Black triangle: gallbladder; White arrow: *T*-tube; Black arrow: drainage tube.

**Figure 8:**
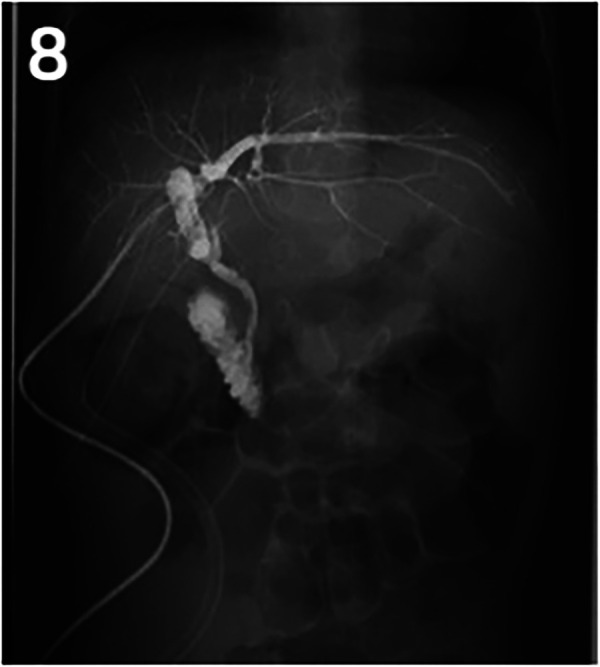
Post-operative *T*-tube cholangiography.

Our center has recently reported on a novel surgical approach known as the bilio-cholecyst anastomosis, inspired by the cholecystoduodenostomy procedure in adults for relieving distal biliary obstruction caused by pancreatic cancer (21). During this procedure, the surgeon separates the gallbladder and gallbladder vessels, prunes the gallbladder bottom to adjust the length and size appropriately, and performs anastomosis of the gallbladder wall and the bile duct (as shown in Figures 7, 8). This approach is designed to make full use of the patient's tissue and preserve the original biliary structure. In patients 2 and 5, we found bile duct injuries at a certain distance from the hepatoportal area, requiring a longer length of intestinal for bilioenteric anastomosis. Due to the potential for tension in the anastomosis direction and the good function of gallbladder, these two patients were selected to undergo the bilio-cholecyst anastomosis procedure. However, this approach requires a suitable location for the gallbladder, good gallbladder function, and the absence of gallstones and other diseases. It is important to balance the risks of surgery against the potential benefits, such as preserving the original biliary structure and utilizing the patient's tissue. It should also be noted that other surgical approaches, such as chronic biloma after right hepatectomy managed with Roux-en-Y biliary cystenterostomy reported by Murphy (22).

In summary, when conservative treatment fails to manage intractable biliary leakage after liver tumor surgery, surgical intervention should be considered. The choice of surgical approach should be guided by the surgeon's experience and the patient's clinical presentation.

In conclusion, intractable bile leakage is a rare and challenging complication that poses significant management difficulties following liver tumor resection in children. Surgery is a reliable treatment for postoperative intractable bile leakage, with various surgical methods available. Among them, bilioenteric anastomosis is considered the most commom technique available. In this study, we report for the first time the feasibility and effectiveness of bilio-cholecyst anastomosis as a surgical approach for the treatment of bile leakage. However, strict indications for surgery should be established to optimize patient outcomes. In addition, infection control and early operation can accelerate the recovery of patients with refractory bile leakage.

## Data Availability

The original contributions presented in the study are included in the article, further inquiries can be directed to the corresponding authors.
